# Lateralised dynamic modulations of corticomuscular coherence associated with bimanual learning of rhythmic patterns

**DOI:** 10.1038/s41598-022-10342-5

**Published:** 2022-04-15

**Authors:** Olivia Morgan Lapenta, Peter E. Keller, Sylvie Nozaradan, Manuel Varlet

**Affiliations:** 1 The MARCS Institute for Brain, Behaviour and Development, Western Sydney University, Penrith, Australia; 2 Institute of Neuroscience, Catholic University of Louvain, Woluwe-Saint-Lambert, Belgium; 3 School of Psychology, Western Sydney University, Penrith, Australia; 4 Present Address: Center for Investigation in Psychology, University of Minho, Braga, Portugal

**Keywords:** Perception, Motor cortex, Human behaviour, Sensorimotor processing

## Abstract

Human movements are spontaneously attracted to auditory rhythms, triggering an automatic activation of the motor system, a central phenomenon to music perception and production. Cortico-muscular coherence (CMC) in the theta, alpha, beta and gamma frequencies has been used as an index of the synchronisation between cortical motor regions and the muscles. Here we investigated how learning to produce a bimanual rhythmic pattern composed of low- and high-pitch sounds affects CMC in the beta frequency band. Electroencephalography (EEG) and electromyography (EMG) from the left and right First Dorsal Interosseus and Flexor Digitorum Superficialis muscles were concurrently recorded during constant pressure on a force sensor held between the thumb and index finger while listening to the rhythmic pattern before and after a bimanual training session. During the training, participants learnt to produce the rhythmic pattern guided by visual cues by pressing the force sensors with their left or right hand to produce the low- and high-pitch sounds, respectively. Results revealed no changes after training in overall beta CMC or beta oscillation amplitude, nor in the correlation between the left and right sides for EEG and EMG separately. However, correlation analyses indicated that left- and right-hand beta EEG–EMG coherence were positively correlated over time before training but became uncorrelated after training. This suggests that learning to bimanually produce a rhythmic musical pattern reinforces lateralised and segregated cortico-muscular communication.

## Introduction

Human movements are spontaneously attracted by rhythmic sensory stimulation. It has been shown that synchronisation even occurs when instructed to avoid it or focusing on another rhythm^1–3^. Notwithstanding, some are more conducive to synchronisation than others, such as musical rhythms or biological rhythms. Based on cortical and subcortical connections between the auditory and motor system, auditory inputs can entrain motor responses, as evidenced by voluntary and involuntary movement synchronisation to auditory rhythms^1,3–6^. This automatic activation of the motor system in response to sensory sequences is a consequence of sensorimotor entrainment^7–9^. Such entrainment supports the perception and production of complex auditory sequences, including music that are coordinated intra-personally (e.g., the two hands of a percussionist) and interpersonally (between ensemble co-performers)^10,11^.

Previous research has provided important insights into the contribution of the motor system in the neural processing of auditory rhythms even without overt movement or the intention to move. Neuroimaging studies demonstrated that motor areas are activated during both rhythm perception and production^12–16^. For example, the basal ganglia and supplementary motor area are activated in beat perception, and such activation is greater for musicians than non-musicians^17^. Magnetoencephalographic (MEG) studies also showed that listening passively to auditory rhythms modulates the amplitude of neural oscillations in the beta range (15–30 Hz)^18,19^ that are involved in movement perception and production^20^. Furthermore, studies using Electroencephalography (EEG) showed selectively enhanced neural activity in response to a rhythmic pattern after a movement training in which participants were trained to move at these specific frequencies marking a metrical interpretation of the rhythm^21^.

Although there is considerable evidence for activity in motor cortices induced by auditory rhythmic stimuli, how these responses transfer at muscular level remains unclear. Not investigated yet in the context of sensorimotor entrainment, cortico-muscular coherence (CMC) is potentially a powerful approach to address this question. Coherence is a correlation measure between frequency domain representations of different signals and CMC is conventionally used as an index of the synchronization between cortical motor regions and associated body muscles^22^. CMC has been proposed to reflect efficiency of neural communication, such that increased coherence between activity of different neural assemblies would reflect their mutual information transfer^23,24^. Although CMC has been investigated within different frequency bands, including theta^25^, alpha^26^, beta^27,28^, and gamma^29,30^, previous research generally focused on the beta range, which shows greater CMC magnitude^24,26,27,31^. CMC is assumed to reflect the coupling between motor activity from the central neural system and muscle discharges^24^. In other words, increased coherence between brain and muscle activity reflects more efficient brain-muscle information transfer. Consistent with this proposal, previous studies showed oscillatory interaction between EEG or MEG and Electromyography (EMG) during sustained contraction^27,32,33^, and that auditory distractors trigger rapid and automatic EMG responses in finger flexor muscles, along with increased CMC in the beta range^34^. Further, it has been argued that insights into muscle synergies gained through the investigation of functional connectivity and motor coordination benefit from complementary information associated with CMC and inter-muscular coherence (IMC) for homologous muscles^35,36^.

In sum, it is well known that audio-motor entrainment is central to rhythm perception and production. In the context of music more specifically, it has been shown that the strength with which the motor system responds, overtly and covertly, is affected by musical expertise^37,38^. Musicians have enlarged somatosensory cortical representations of the fingers involved in playing their instrument, and also larger auditory cortical areas responding to music-related acoustic stimuli when compared to non-musicians^39^. Considering that precise temporal audio-motor coordination and integration are necessary to perceive and produce rhythms in synchrony^38,40^, one could suggest a bidirectional influence where music training reinforces neural pathways related to sensorimotor entrainment. In fact, a previous fMRI study demonstrated that auditory-only and motor-only (music related) tasks engage the secondary motor (pSMA, PMv, PMd) and auditory cortex, respectively, and that such transmodal activity is significantly stronger in skilled pianists when compared to non-musicians^41^. Lahav and colleagues^42^ demonstrated similar audio-motor transmodal activations in musically naive subjects. Specifically, after brief training at playing a piano piece by ear, passive listening to the trained music resulted in activation of additional frontoparietal motor-related regions^42^. Finally, a MEG study showed greater music-elicited mismatch negativity (a negative event-related potential that is generated by the brain's response to changes in auditory stimulation exceeding a certain limit roughly corresponding to the behavioural discrimination threshold^43^) in the auditory cortex after sensorimotor-auditory training compared to auditory training alone^44^. This data demonstrates the functional connection between auditory and motor systems, and that sensorimotor-auditory training promotes plasticity within the auditory cortex to a greater extent compared to auditory training alone, thus suggesting that learning predictions about upcoming musical events actively involves the motor system^44^.

Therefore, we propose that learning how to play an auditory rhythmic pattern where the right and left hands are used to play two different sounds of the pattern would promote specific entrainment of cortico-muscular communication with right and left hand muscles while listening to the learnt pattern. In order to test this hypothesis, we exposed non-musicians to a rhythmic pattern composed of two sounds (low-pitch and high-pitch percussion sounds) while concurrently recording EEG and EMG activity from two muscles at right and left hands and arms. Recordings were made before and after a training session where participants learnt how to play the rhythmic pattern guided by visual cues by pressing the force sensors with their left or right hands in order to produce the low- and high-pitch sounds, respectively. We evaluated short-term effects of audiomotor rhythmic learning in laterality-specific motor entrainment at cortical and muscular levels by means of the magnitude of brain oscillations, and of cortico-muscular and inter-muscular coherence, in the beta frequency range. We expected more beta desynchronisation and higher beta CMC when listening after training, indicating that learning the rhythm promotes greater entrainment. We also expected that entrainment would become more specific after training, as reflected by selective modulation of the beta cortico-muscular communication for the right and left hands while listening, according to the sounds that they have learnt to play. In order to examine whether bimanual training promotes lateralised entrainment and beta cortico-muscular modulations, we performed between-hand correlations to test integrated vs. segregated pattern before and after training, respectively.

## Methods

### Participants

Thirty-two subjects were invited to participate in the experiment, which was performed in accordance with the Declaration of Helsinki and approved by the Human Research Ethics Committee at Western Sydney University (reference #H10487). All participants gave their written informed consent and complied with the following criteria: age between 18 and 50 years, right-handed, normal or corrected-to-normal visual acuity, and no known past or current auditory impairment, psychological or psychiatric disorders, and central nervous system injury, with no formal or informal musical training. Five participants were excluded from the initial sample as their EEG and/or EMG signals presented abnormal excessive noise and artifacts due to loose EEG ground electrodes and/or EMG electrodes for more than 50% of the listening trials. Therefore, the final sample comprised 27 participants (12 male) aged between 19 and 41 years (M = 30.63, SD = 4.32).

### EEG and EMG recordings

Electrophysiological (EEG) and Electromyographic (EMG) data were recorded with a BioSemi ActiveTwo system (BioSemi, The Netherlands). EEG was recorded by means of 64 active Ag–AgCl electrodes placed on the scalp according to the International 10/20 system. EMG was recorded using 8 external Ag–AgCl channels. EMG electrodes were positioned over the right and left First Dorsal Interosseous (FDI) and Flexor Digitorum Superficialis (FDS)^32,45^ following a classic belly-tendon montage. Both muscles are involved in the pincer movement needed to hold the sensor and playing the sequence. Whereas FDI is more superficial, facilitating recording, previous studies showed CMC effects on FDS^45^, therefore we opted to include both. EEG and EMG data were recorded at a sampling rate of 2048 Hz, and stored for offline analysis.

### Stimuli and procedure

The sound of a single strike from a *surdo* and a *bongo* (percussion instruments) were used to create a rhythmic pattern composed of low-pitch (approximately 90 Hz fundamental frequency) and high-pitch sounds (approximately 430 Hz fundamental frequency), respectively, each with 500 ms duration consisting of a sharp onset (~ 10 ms rise time) for both sounds followed by a rapid decay for the high-pitch sound (~ 60 ms) and a slower decay (~ 350 ms) for the low-pitch sound (see Fig. A in Supplementary Material for further details). The tones were equalised for perceived loudness following the Cambridge loudness model^46^ also applied in similar research^47,48^ using Matlab (The MathWorks Inc., Natick, MA, USA). The sounds were then arranged in a 5 s pattern composed of “XO.OX.O.O.XX” where “X” represents the low-pitch sounds (i.e., *surdo*); “O” the high-pitch sounds (i.e., *bongo*); and “.” a silence period of 250 ms.

The pattern was presented in two categories of trials: (1) *Listening* trial with a total duration of 2 min, composed only of auditory stimuli, containing 24 concatenated repetitions of the pattern (before presentation of the stimuli, participants were explicitly informed that the rhythm was repeated over time); and (2) *Training* trial with a total duration of 1 min. The training trial started with 20 s containing 4 repetitions of the pattern accompanied by visual stimuli, consisting of dots that flashed at the left-hand side of the screen for low-pitch sounds and at the right-hand side of the screen for high-pitch sounds. The initial 20 s of training trial were directly followed by 40 s without visual stimuli, where participants were instructed to continue producing the pattern by pinching the force sensors, with the left sensor producing the low-pitch sound (i.e., *surdo*) and right sensor producing the high-pitch sound (i.e., *bongo*), while receiving auditory feedback of their produced rhythm. Participants were instructed to adjust their pressure timings to best match the previously listened pattern. Auditory stimuli were delivered binaurally via insert earphones (ER‐1, Etymotic Research, Elk Grove Village, IL, USA) at a comfortable but clearly audible intensity that was the same for all participants. Visual stimuli were red (RGB: 255,0,0) dots with 5 cm of diameter presented on a VIEWPixx monitor (VPixx Technologies, Saint-Bruno, Canada) with a 120 Hz refresh rate and appeared for 14 frames (i.e., about 117 ms).

Upon arrival, all participants were informed about the procedure and provided written informed consent. After EEG and EMG preparation, participants were asked to sit comfortably on a chair at a distance of approximately 60 cm from the monitor. They were given two pressure sensors to hold between the index finger and thumb. The force sensors consisted in wide bar load cells (HTC-Sensor TAL201, Colorado, USA) connected to an Arduino Duemilanove board (Arduino, Ivrea, Italy) via an amplifier shield (Load Cell/Wheatstone Amplifier Shield, RobotShop, Mirabel, Quebec, Canada). Participants then received verbal and on-screen instruction to pinch with both hands at their maximum force in three consecutive pre-trials of 5 s. The maximum force recorded in each of the three 5 s pre-trials were averaged and used in the following experimental trials.

Before each Listening trial (both pre- and post-training), two feedback bars on the right and left-hand sides of the screen were presented and participants had to adjust the pressure applied to the sensor to match 10% of their maximum force. Trials only began once the exerted force for both hands equated 10% ± 7% of the maximum force. Although the feedback bars disappeared upon the beginning of the trial, participants were instructed to maintain this constant pressure during the entire trial. Participants were only instructed to maintain the pressure as steady as possible and no other instructions related to the auditory stimuli were given in these trials.

For the Training trials, participants were instructed to play along with the auditory pattern by pinching the force sensors with the help of the right and left visual stimuli flashing to indicate which sensor had to be pinched. They were warned that after four repetitions the visual cue would disappear and that they should keep playing the same rhythmic pattern.

**Figure 1:**
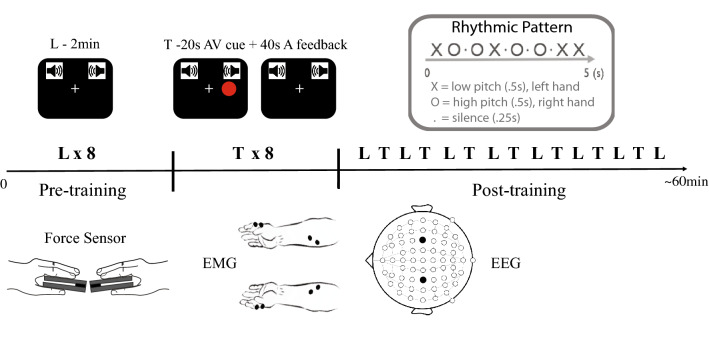
Experimental Design. Sound was delivered binaurally. Force sensors were held between index finger and thumb. EMG was recorded from left and right FDI and FDS muscles. EEG was recorded with 64 channels and analyses were conducted using C3 and C4 data. Listening (L) trials before and after the 8 training (T) trials were considered as pre and post-training, respectively.

The task was elaborated to contain 8 trials lasting 2 min each in a pre-training Listening condition, and 8 trials lasting 2 min each in a post-training Listening condition. To learn how to produce the pattern, 8 consecutive Training trials of 1 min each were presented after the pre-training condition and 7 additional trials of 1 min each were presented in alternation with the listening trials in the post-training condition (see Fig. 1) in order to reinforce learning and maintain participants' engagement. The experiment was programmed in C++ on a MacBook Pro laptop (Apple Inc., California, USA). Participants took roughly 1 h to complete the task, and the EEG and EMG preparation varied between 30 and 50 min. Therefore, the experiment was in total within 1.5–2 h long.

### Training data processing and analysis

To evaluate if training was effective at allowing participants to learn how to play the pattern, we computed, for each hand and trial, the absolute time difference between the inter-sound intervals the participant was supposed to perform and the ones the participant actually produced. Following this, we averaged the mean absolute time difference of the right and left hands for each trial. Outliers were identified as any data point more than 1.5 interquartile range (IQR) below the first quartile or above the third quartile. To evaluate if task performance improved significantly across training, we performed a paired-sample t-test contrasting the first and last trial mean absolute time difference.

### EEG and EMG data processing

Data was processed using the Fieldtrip toolbox for EEG/EMG analysis^49^ in the MatLab software environment (The MathWorks, Natick, USA). Listening data was segmented in epochs of 120 s from the beginning of the sound pattern. EEG channels with excessive noise were interpolated by the mean of surrounding channels; a maximum of 6 EEG channels per participant was interpolated (M = 1.55, SD = 1.39 of interpolated channels). Trials were considered invalid and rejected when visual inspection detected (1) peaks in EMG due to movement or loss of contact or (2) large waves in EEG due to issues with the reference or ground channels. Each participant had maximum 3 rejected trials per condition (M = 0.11, SD = 0.31 eliminated trials due to poor EEG quality; M = 0.29, SD = 0.76 eliminated due to poor EMG quality). Independent component analysis was performed using the FASTICA algorithm, as implemented in Fieldtrip, to identify and remove eye blink and lateralised eye movement artefacts. The average number of ICA components removed per participant was of 1.93 (SD = 0.60). A 0.1 Hz and 10 Hz high-pass filter was applied for EEG and EMG, respectively. We filtered EMG signals below 10 Hz to avoid possible movement-related interferences, which is a common approach in EMG and cortico-muscular (EEG–EMG and MEG–EMG) coherence studies focusing on beta and faster frequencies^27,45,50,51^. Both EEG and EMG data were notch filtered to remove 50 Hz power contamination and its harmonics. EEG data was re-referenced to the average of all EEG channels, and each EMG belly channel was referenced to the corresponding tendon channel and then rectified^52^. All data was downsampled to 1000 Hz and stored for further analyses.

Time–frequency analyses, as implemented in Fieldtrip, were conducted on EEG and EMG channels to retrieve oscillatory activity within 10–50 Hz and compute EEG–EMG and EMG–EMG coherence. A fast Fourier transform (FFT) was computed on 250 ms window sliding by 10 ms steps, resulting in power spectra and cross-spectra with a 4 Hz frequency resolution. A multitaper method based on Slepian sequences as tapers was applied, for optimal spectral concentration of energy within a range of frequency space^53^, to compute the power and cross-spectra over time^27,34,45^. Three tapers in total were used, leading to a spectral smoothing of ± 6 Hz. The time–frequency maps obtained for each Listening condition (i.e., pre- and post-training) were then reshaped in 24 windows of 5 s long (i.e. the length of the sound pattern), thus corresponding to the 24 repetitions of the sound pattern for each trial. Next, we computed the averaged power and coherence in the beta (16–36 Hz) frequency range over the 5 s window. For the beta band, we selected the 16–36 Hz range that contains both low and high beta ranges while avoiding high alpha^45^, which is in line with previous studies that reported beta oscillations, as well as beta cortico-muscular coherence, ranging from 15 to 35 Hz^32,45,51,54^. Further, this range was selected to capture the range of frequencies at which EEG–EMG coherence occurred across all participants (see Fig. B in Supplementary Material showing the mean and variability in the frequency range in which coherence was observed across participants). Channels C3 and C4 were used to examine cortical activities related to the right and left hand, respectively^55,56^. CMC was computed between C3 and right limb muscles, and between C4 and left limb muscles. The selection of C3 and C4 electrodes was based on the extensive literature showing C3 and C4 as main key loci for sensorimotor activity and CMC^50,55^, as seen in Fig. 4 showing maximum grand-averaged CMC at these electrodes for both FDI and FDS muscles. IMC was computed between right and left limb homologous muscles. All computations (i.e., EEG and EMG power, CMC and IMC) were performed for listening trials before and after training.

Broadband EMG responses were also examined to determine whether there was any modulation in participants’ muscular activity induced by the stimulus presentation despite being instructed to maintain a constant finger pressure. The envelope of the preprocessed EMG signals (i.e., 10 Hz high-pass filtered and rectified) was extracted using a Hilbert transform to capture global changes in the amplitude of muscular activity^27,45^. The envelopes of the different trials were then reshaped in windows of 5 s corresponding to the sound pattern and averaged together.

### EEG and EMG power analysis

EEG and EMG power data and EMG broadband data from all time-bins in response to the sound pattern were first averaged. We then performed repeated measures analyses of variance (rmANOVAs) to evaluate changes in EEG power, considering Training (Pre vs. Post) and Electrode (C3 vs. C4) as factors, and averaged beta power as the dependent variable. Analogously, for EMG we considered Training (Pre vs. Post), Muscle (FDI vs. FDS) and Hand (Right vs. Left) as factors, and averaged beta power, as well as broadband amplitude (i.e., envelope of the rectified EMG extracted with the Hilbert transform), as dependent variables.

We also computed the correlation coefficient between the EEG beta power at C3 and C4 over the time of the 5 s pattern, and between the right and left FDI/FDS EMG power within the beta band and the broadband EMG before and after training for each participant to examine the degree of co-activation between the right and left hands at cortical and muscular levels.

Further, Bayesian equivalent tests were performed to report statistical evidence using Bayes factors (BFs), BF_10_ for paired sample comparisons and correlational analysis and BF_incl_ for ANOVAs denoting the level of evidence for the alternate hypothesis (non-signed difference), and the inclusion of a specific parameter in a model (ANOVA), respectively.

### Cortico-muscular and intermuscular coherence analyses

As for the power analysis, we averaged all time-bins of the computed beta CMC for the left and right-hand muscles at their corresponding cortical representation (C4 and C3, respectively). Following, rmANOVA was performed on the mean of CMC considering Training (Pre vs. Post), Muscle (FDI vs. FDS) and Hand (Left vs. Right) as factors.

To further examine the degree of integration/segregation between the two hands (i.e., if the bimanual action is elaborated as an integrated response of complementary information or segregated into different specialised modules^57^) and how it is modulated by training, we also computed the correlation coefficient between left and right FDI/FDS muscles on CMC data. Correlation coefficients were submitted to a rmANOVA with the factors Muscle (FDI vs. FDS) and Training (Pre vs. Post).

To evaluate changes in coherence between the muscles of the left and right hands before and after training, we averaged all time-bins of the computed IMC within the beta range and performed a rmANOVA considering muscle (FDI vs. FDS) and Training (Pre vs. Post) as factors.

Further, Bayesian equivalent tests were performed to report statistical evidence using Bayes factors (BFs), BF_10_ for paired sample comparisons and correlational analysis and BF_incl_ for ANOVAs denoting the level of evidence for the alternate hypothesis (non-signed difference), and the inclusion of a specific parameter in a model (ANOVA), respectively.

## Results

### Training effect on performance

Data of the first trial showed a uniform and fairly symmetrical distribution with a kurtosis value of − 0.773 (SE = 0.872) and a skewness value of 0.071 (SE = 0.448). Data for the last trial showed a uniform but moderately positively skewed distribution, with a kurtosis value of − 0.415 (SE = 0.872) and a skewness value of 0.994 (SE = 0.448). No outliers were found.

**Figure 2:**
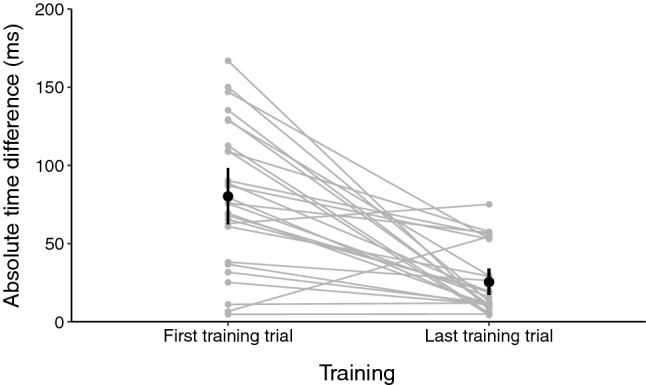
Absolute time difference between the inter-sound intervals that participants were instructed to perform and the intervals that participants actually produced before and after training. The black dot and bars represent the mean and confidence interval. Grey dots represent averaged data for individual participants. The significant reduction on the absolute time differences throughout training indicate successful training with higher production precision gained through practice.

A paired-sample t-test comparing the mean absolute time difference of the first and last training trials indicated a significant decrease after training (t_26_ = 5.671, *p* < 0.001, d = 1.098), showing that participants learnt to produce the bimanual pattern (Fig. 2).

### EEG beta power

The ANOVA on the time-averaged EEG beta power considering Training (Pre vs. Post) and Electrode (C3 vs. C4) as factors revealed a significant main effect of Electrode, F_1,26_ = 6.155, *p* = 0.020, *η*_*p*_^2^ = 0.191, BF_Inc_ = 4.605, showing higher power at C3 (M = 1.040 SE = 0.149) compared to C4 (M = 0.887 SE = 0.110). However, topographic maps suggest that source for such modulation was unspecific to motor areas and seems to originate from more frontal activity, and potentially residual of eye movements (see Fig. C in Supplementary Material). No effect of Training (*p* = 0.211, BF_Inc_ = 0.541) nor for Electrode × Training interaction (*p* = 0.916, BF_Inc_ = 0.439) was observed.

The t-test between Pre and Post training on correlation coefficients between EEG beta power at C3 and C4 indicated no difference in the interhemispheric correlation for beta power over the time of the 5 s sound pattern, t_26_ = 0.557, *p* = 0.582, d = 0.107, BF_10_ = 0.235.

### EMG power

#### Beta

The ANOVA on EMG beta power considering Muscle (FDI vs. FDS), Hand (Right vs. Left), Training (Pre vs. Post) as factors revealed a main effect of Muscle, F_1,26_ = 39.85, *p* < 0.001, *η*_*p*_^2^ = 0.605, BF_Inc_ = 1.66e+13, showing greater power for FDI (M = 240.282 SE = 37.558) compared to FDS (M = 3.540 SE = 0.682). No effect of Hand (*p* = 0.927, BF_Inc_ = 0.068) or Training (*p* = 0.859, BF_Inc_ = 0.065), nor for the Muscle × Training interaction (*p* = 0.877, BF_Inc_ = 0.059), Muscle × Hand (*p* = 0.867, BF_Inc_ = 0.060), Training × Hand interaction (*p* = 0.743, BF_Inc_ = 0.012) and Muscle × Hand × Training (*p* = 0.738, BF_Inc_ = 8.06e−4) interaction was observed.

The ANOVA on the correlation coefficient between the right and left FDI/FDS EMG power within the beta band showed no significant effects for Muscle (*p* = 0.738, BF_Inc_ = 0.150), Training (*p* = 0.255, BF_Inc_ = 0.369) or Muscle Training interaction (*p* = 0.366, BF_Inc_ = 0.087).

#### Broadband

The ANOVA on EMG broadband amplitude (i.e., envelope of the EMG signal) considering Muscle, Hand and Training as factors indicated a main effect of Muscle, F_1,26_ = 149.213, *p* < 0.001, *η*_*p*_^2^ = 0.852, BF_Inc_ = 3.22e+15. This effect shows higher amplitude for FDI (M = 92.952 SE = 6.871) than FDS (M = 12.843 SE = 1.009). This analysis did not reveal any other significant effects for Training (*p* = 0.531, BF_Inc_ = 0.0845), Hand (*p* = 0.825, BF_Inc_ = 0.070), Muscle × Training (*p* = 0.679, BF_Inc_ = 0.074), Muscle × Hand (*p* = 0.499, BF_Inc_ = 0.074), Training × Hand (*p* = 0.280, BF_Inc_ = 0.014), Muscle × Training × Hand (*p* = 0.308, BF_Inc_ = 0.001).

The ANOVA on the correlation data between the right and left FDI/FDS EMG broadband power considering the factors Muscle (FDI vs. FDS) and Training (Pre vs. Post) yielded no significant effects for Muscle (*p* = 0.595, BF_Inc_ = 0.176), Training (*p* = 0.764, BF_Inc_ = 0.145) or Muscle × Training interaction (*p* = 0.739, BF_Inc_ = 0.039).

### Cortico-muscular coherence

The ANOVA on the time-averaged beta coherence considering Muscle, Hand and Training as factors revealed a main effect of Muscle, F_1,26_ = 9.673, *p* = 0.004, *η*_*p*_^2^ = 0.271, BF_Inc_ = 54.510. Specifically, coherence for FDI (M = 0.077 SE = 0.008) was higher than for FDS (M = 0.068 SE = 0.007) muscle. No significant effects were found for Electrode (*p* = 0.821, BF_Inc_ = 0.166), Training (*p* = 0.241, BF_Inc_ = 0.252), Muscle × Electrode (*p* = 0.613, BF_Inc_ = 0.237), Muscle × Training (*p* = 0.448, BF_Inc_ = 0.209), Electrode × Training (*p* = 0.384, BF_Inc_ = 0.214), Muscle × Electrode × Training (*p* = 0.327, BF_Inc_ = 0.280). Note that two control analyses, (1) on CMC in the 16–32 Hz range, excluding higher beta frequencies and (2) considering left (C1, C3, C5, FC3, CP3) and right (C2, C4, C6, FC4, CP4) electrode clusters, indicated similar results (see SM for further details).

**Figure 3:**
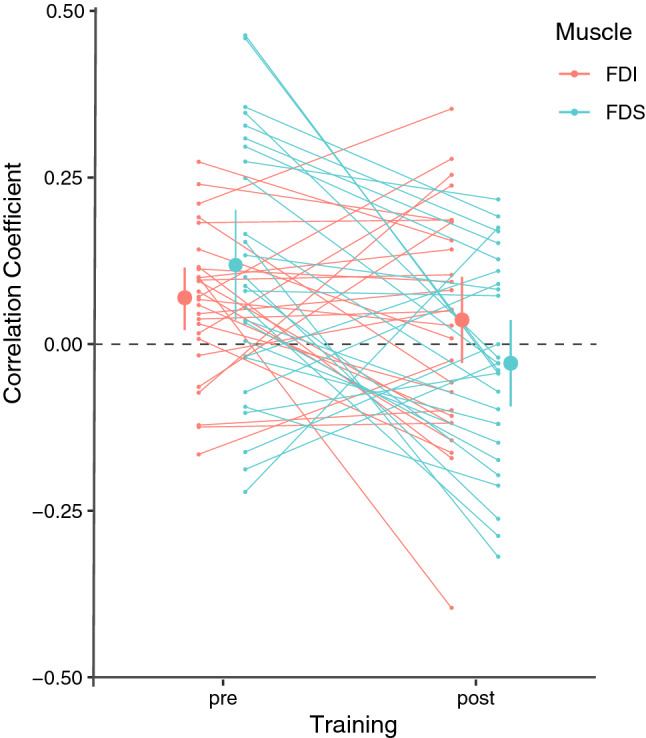
Mean and confidence interval of the correlation coefficient between right and left hand beta CMC for FDI (pink) and FDS (blue) muscles before and after training (represented by larger dots and bars). Smaller dots represent data for individual participants. The left and right hand FDS muscle that were positively correlated pre-training became uncorrelated post-training, indicating segregation of left and right hand after training.

**Figure 4:**
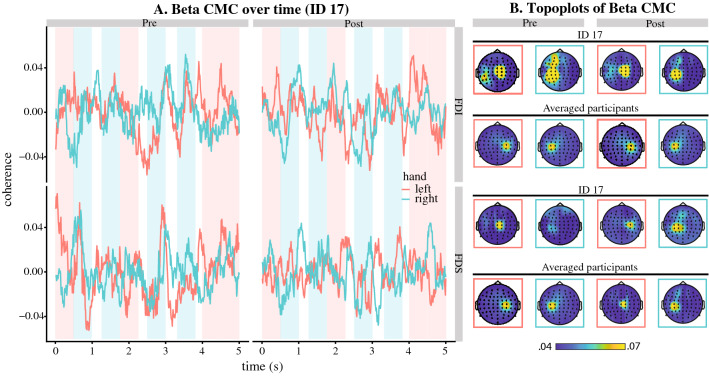
Cortico-muscular coherence in the beta band (16–36 Hz). Panel (**A**) shows demeaned beta CMC data throughout the rhythm pattern for a representative participant (ID 17) before (Pre) and after (Post) training for the right hand-C3 (blue) and left hand-C4 (pink) for FDI (first dorsal interosseus) and FDS (flexor digitorum superficialis) muscles. The shaded colours represent the low-pitch (pink) and high-pitch (blue) sounds that were learnt to be played using left and right hand, respectively. Panel (**B**) represents the topoplots of the same participant and the average of all participants for beta CMC for FDI and FDS muscles before (Pre) and after (Post) training.

The ANOVA on the correlation coefficients between left and right FDI/FDS coherence with C4 and C3, respectively, considering Muscle and Training as factors yielded a significant main effect of Training, F_1,26_ = 8.332, *p* = 0.008, *η*_*p*_^2^ = 0.243, BF_Inc_ = 8.195 and Muscle × Training interaction, F_1,26_ = 4.410, *p* = 0.046, *η*_*p*_^2^ = 0.145, BF_Inc_ = 0.863. Bonferroni corrected post hoc analysis revealed a significant difference between Pre- and Post-training conditions for the FDS muscle, t_26_ = 3.259, *p* = 0.019, d = 0.627, BF_10_ = 12.648, but not FDI, t_26_ = 0.828, *p* = 1, d = 0.159, BF_10_ = 0.279. As illustrated in Fig. 3, one sample t-tests indicated that both FDI, t_26_ = 2.985, *p* = 0.006, d = 0.575, BF_10_ = 7.093 and FDS, t_26_ = 3.005, *p* = 0.006, d = 0.578, BF_10_ = 7.395, correlation coefficients were significantly above zero in the Pre-training condition and did not differ from zero in the Post-training condition (FDI t_26_ = 1.029, *p* = 0.313, d = 0.199, BF_10_ = 0.329; FDS t_26_ = − 0.881, *p* = 0.386, d = 0.170, BF_10_ = 0.290). Beta CMC for the left and right hands were positively correlated before training whereas they were uncorrelated after training. Beta CMC for the right and left hands over the length of the pattern for one representative participant is illustrated in Fig. 4A, along with topographic maps of the same participant and averaged across all participants (Fig. 4B). No main effect was observed for Muscle (*p* = 0.866, BF_Inc_ = 0.318).

### Intermuscular coherence

The ANOVA on the averaged IMC considering Muscle and Training as factors indicated a main effect of Muscle, F_1,26_ = 9.686, *p* = 0.004, *η*_*p*_^2^ = 0.271, BF_Inc_ = 16.295, also showing higher coherence for FDI (M = 0.046 SE = 0.002) than FDS (M = 0.042 SE = 0.001). The ANOVA did not yield any other significant effects for Training (*p* = 0.839, BF_Inc_ = 0.177) or Muscle × Training interaction (*p* = 0.731, BF_Inc_ = 0.177).

## Discussion

Here we investigated brain and muscular activity, and the synchronisation between these two signals by means of EEG–EMG cortico-muscular coherence in the beta band, before and after learning a bimanual rhythmic pattern consisting of different pitched sounds produced by the two hands. The proposed training effectively improved task performance, reflecting that participants learnt how to play the bimanual rhythmic pattern. Importantly, learning the bimanual rhythmic pattern affected cortico-muscular communication during passive listening. Specifically, our results show that dynamic modulations in right- and left-hand beta band CMC that were positively correlated before training were uncorrelated after training. This change after training is likely due to a lateralised and segregated activation pattern elicited in accordance with the hand recruited to produce each particular sound in the sequence.

Selective brain processing shaped by movements has been previously demonstrated with EEG, showing that periodic body movement performed on a rhythmic pattern leads to enhanced brain response at these periodicities corresponding to the movement when participants subsequently listen to the rhythmic pattern without moving^21^. Further, Perez et al.^58^ showed that visuo-motor training increases CMC between muscle and cortical representation specifically involved in training. Our study extends these findings by showing decreased correlation between the CMC of right and left upper limb muscles and their cortical representations after training in a condition where participants are listening to the auditory signals they learnt to produce with hand movements. We speculate that this effect occurs after training because of spontaneous alternating recruitment of the cortico-muscular connections involved in producing each different sound even if participants were simply passively listening to them. This is in line more generally with previous research showing that CMC of one hand decreases during concurrent movement of the other hand due to divided attention^50^. This is also in accordance with previous research that found changes in auditory-motor interactions with musical expertise^17,38,41^, extending this research by demonstrating how quickly changes in cortico-muscular interactions elicited by auditory rhythm can occur with training. In turn, as auditory training per se affects CMC, our study is limited by the fact that we do not have a control group submitted solely to the auditory rhythm presentation. Considering that our effects were specifically related to lateralisation, it is not likely to be a result of auditory exposure alone. Pure auditory training would be expected to increase general CMC without specifically indexing a decrease of correlation between lateralised brain-muscle engagement. However, further studies are needed to clarify if specific sound pitches, in non-musicians, could be more likely to engage right or left hand muscles and therefore an auditory stimulation effect alone cannot be completely excluded.

In order to control for the observed CMC effects and the possibility that they would be related to individual EEG or EMG modulations, we also analysed beta power oscillations in both EEG and EMG data. The correlation of EEG beta amplitude over time between C3 and C4 did not change after training. The correlation between left and right EMG did not change either for both FDI and FDS muscles, suggesting that lateralised CMC responses after training did not originate from more lateralised responses at cortical and/or muscular levels.

Interestingly, beta inter-muscular coherence (IMC) between homologous right and left hand muscles did not show any effect of training. The lack of evidence for decreased synchronisation in IMC between the left and right hand muscles after training as opposed to the decreased synchronisation observed in CMC suggests that distinct processes underpin cortico-muscular and bilateral motor unit synchronisation^59^. Specifically, EEG–EMG and EMG–EMG coherence display dissimilar temporal and frequency profiles, with peak IMC occurring earlier and in lower frequencies compared to CMC^59^. Furthermore, Carr et al.^60^ found no evidence for cross-correlations between several homologous upper-limb muscles, suggesting that those may be co-activated voluntarily but often act independently. Our results show that bimanual training requiring the segregation, i.e., the capacity to separate information into modules that perform specialised computations^57^, of the left and right hands selectively modulates the processes underlying cortico-muscular communications independently from those underlying inter-muscular synchronisation. Our findings are broadly consistent with research on whether bimanual polyrhythm production involves integrated or independent timing control across the two hands^61,62^. Previous work has shown that this varies as a function of tempo and expertise^63^. Of relevance to the present results, experts (i.e., trained musicians) are able to employ flexible approaches, and can achieve high level performances with independent control^64^. Our pattern was elaborated to have a level of difficulty suitable for novice participants and to have the same number of stimuli for the left and right hands. It is possible that different tones, as well as different sound and silent durations would promote entrainment to a lesser or greater extent. Still, our study evaluated only one rhythmic pattern as the main goal was to evaluate the learning effects on cortico-muscular coupling of the specific hand involved in producing the sound, and decoupling of the hand not involved in producing the sound. One interesting avenue for future studies would be to investigate if and which different rhythmic characteristics impact the entrainment and learning of the rhythmic pattern, and also if using a more challenging pattern would require even more segregation. Furthermore, we attributed the low and high pitch to the left and right hand, respectively, and possible hand-pitch effects were not investigated. Counterbalancing the hand-pitch combinations in future research could also be interesting for further control as well as investigating the role of each hand separately.

The results also revealed that beta CMC was greater for FDI compared to FDS muscles. Differential EEG–EMG coherence for different muscles has been previously reported, and has been attributed to variations in the location and orientation of corticospinal neurons^50^. In particular, larger cortical representations of the muscles, as well as the robustness and superficiality of the muscle itself, likely allow better recordings due to superior signal-to-noise ratio, and thus, stronger CMC^50^. EMG analyses also showed larger amplitude in FDI compared to FDS in both beta and broadband data. Recording of FDI might have been facilitated by the fact that FDI is more superficial compared to FDS, which could partially explain the greater CMC for this muscle. However, such EMG amplitude differences between FDI and FDS is unlikely to explain the correlation effects reported above, which were actually stronger for FDS. Differences between EMG power peak and EEG–EMG coherence peak are not uncommon and have been previously reported during isometric contraction tasks^50^. In fact, some have argued that these measures are dissociated^65,66^, and that coherence relies on the EEG–EMG amplitude ratio and the phase differences between them rather than the amplitudes of EEG or EMG signals alone^67^.

Finally, these results focused on the beta band since its relevance has been extensively demonstrated in cortico-muscular literature. Still, although CMC is much weaker in other frequency bands, there are studies suggesting that these bands might also play a role^68^, and therefore, investigating CMC at other frequencies such as in alpha and gamma range is a potential avenue for future research.

To conclude, this study found that the correlation between right- and left-hand beta CMC is modified by motor training of a bimanual rhythmic pattern. To our knowledge this is the first report of significant effect of learning on the cross-correlation between left and right CMC when listening to a rhythmic pattern without movement or intention to move. It is likely that motor cortex entrainment was shaped according to the bimanual training that led to the automatic association of the low-pitch with the left hand and high-pitch with the right hand, resulting in lateralised CMC responses even during passive listening. With no effect of training observed for homologous muscle coherence, our results also indicate distinct underlying processes for CMC and IMC that can be separately modified by motor learning. Plasticity in the functional configuration of the motor system may thus underpin the perception and production of complex auditory sequences, with the coupling between cortical and muscular activity providing an index of audio-motor entrainment and musical and rhythmic skills.

## Supplementary Information

Supplementary Information.
