# Supplementary material for: Lateralised dynamic modulations of corticomuscular coherence associated with bimanual learning of rhythmic patterns
Source: Sci Rep. 2022 Apr 15;12:6271. doi: 10.1038/s41598-022-10342-5 (PMC9012795; doi:10.1038/s41598-022-10342-5)

SUPPLEMENTARY MATERIAL

1. Table 1 shows the Mean and SD of EEG power within the Beta range for C3 and C4 before and after training.

Electrode	Training	Mean	SD
C3	Pre	0.995	0.743
	Post	1.085	0.856
C4	Pre	0.844	0.574
	Post	0.929	0.611

2. Table 2 shows the Mean and SD of EMG beta power for the FDI and FDS muscles for the left and right hand before and after training.

Muscle	Training	Hand	Mean	SD
FDI	Pre	Left	243.57	248.82
		Right	229.92	270.28
	Post	Left	242.85	269.25
		Right	244.78	247.51
FDS	Pre	Left	2.35	2.93
		Right	4.23	5.74
	Post	Left	2.96	3.33
		Right	4.63	5.25

3. Table 3 shows the Mean and SD of the broadband EMG amplitude for the FDI and FDS muscles for the left and right hand before and after training.

Muscle	Training	Hand	Mean	SD
FDI	Pre	Left	95.5	45.08
		Right	87.9	41.79
	Post	Left	96.0	54.16

		Right	96.3	48.01
FDS	Pre	Left	11.4	4.84
		Right	13.3	7.26
	Post	Left	12.4	5.98
		Right	14.3	7.15

4. Table 4 shows the Mean and SD for the Cortico-muscular Coherence within the Beta band between the FDI and FDS muscles and the C3 and C4 electrodes before and after training.

Muscle	Electrode	Training	Mean	SD
FDI	C4	Pre	0.0759	0.0415
		Post	0.0791	0.0480
	C3	Pre	0.0752	0.0386
		Post	0.0791	0.0444
FDS	C4	Pre	0.0694	0.0383
		Post	0.0692	0.0389
	C3	Pre	0.0654	0.0340
		Post	0.0693	0.0398

5. Table 5 shows the Mean and SD for the Inter-muscular Coherence within the Beta band between the FDI and FDS muscles before and after training.

Muscle	Training	Mean	SD
FDI	Pre	0.0459	0.01054
	Post	0.0465	0.00844
FDS	Pre	0.0421	0.00739
	Post	0.0421	0.00850

6. Table 6 shows the Mean and SD for the averaged mean absolute time difference of the right and left hands for the first and last training trials.

	**Mean**	**SD**
First Trial	80.2	45.4
Last Trial	25.4	21.0

7. Table 7 show results from the rmANOVA control analysis performed on the CMC in the 16-32 Hz range (excluding the higher beta frequencies) considering Training (Pre vs Post), Muscle (FDI vs FDS) and Hand (Left vs Right) as factors.

CMC	**F**	**p**	**η^2_p**	**BF_{Inc}**
Muscle	10.792	0.003	0.293	46.967
Training	0.138	0.714	0.005	0.078
Hand	0.015	0.905	0.001	0.068
Muscle ✻ Hand	0.088	0.769	0.003	0.062
Muscle ✻ Training	3.256	0.083	0.111	0.011
Training ✻ Hand	0.418	0.524	0.016	0.013
Muscle ✻ Training ✻ Hand	0.617	0.439	0.023	0.002

8. Table 8 show results from the rmANOVA control analysis performed on the CMC (16-36 Hz), computed using the left (C1, C3, C5, FC3, CP3) and right (C2, C4, C6, FC4, CP4) electrode clusters, considering Training (Pre vs Post), Muscle (FDI vs FDS) and Hand (Left vs Right) as factors.

CMC	**F**	**p**	**η^2_p**	**BF_{Inc}**
Muscle	7.053	0.013	0.213	9.596
Training	1.645	0.211	0.060	0.138
Hand	0.392	0.537	0.015	0.139
Muscle ✻ Hand	0.360	0.554	0.014	0.108
Muscle ✻ Training	0.193	0.664	0.007	0.102
Training ✻ Hand	0.390	0.538	0.015	0.032
Muscle ✻ Training ✻ Hand	1.772	0.195	0.064	0.002

9. Figure A illustrates the sounds used in the experiment. The upper panel depicts the high pitch sound wave and the lower panel depicts the low pitch sound wave.

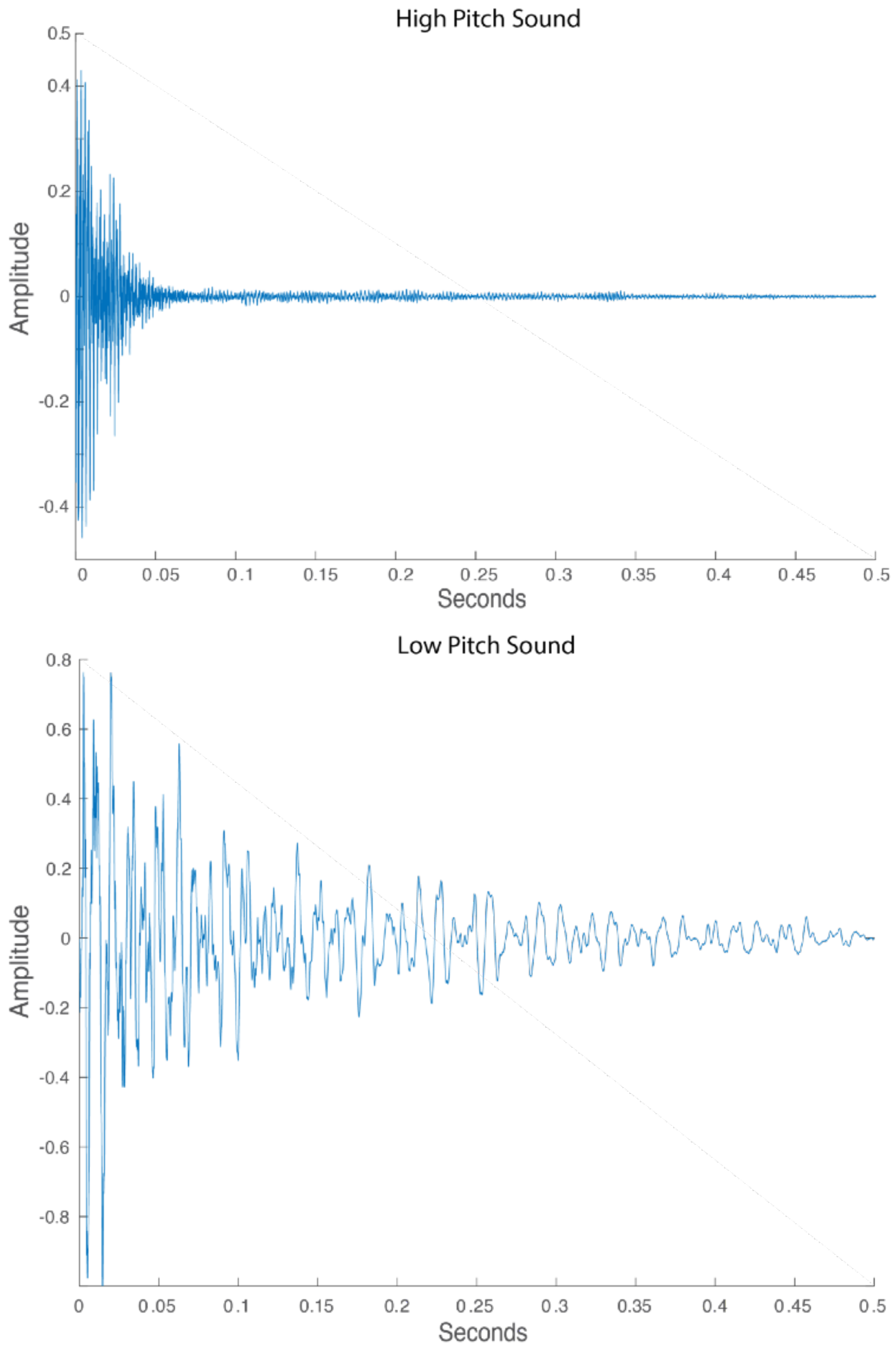

10. Figure B shows CMC averaged across all listening trials for both FDI and FDS muscles as a function of frequency. The black line shows the average of all participants and the blue lines represent each individual curve.

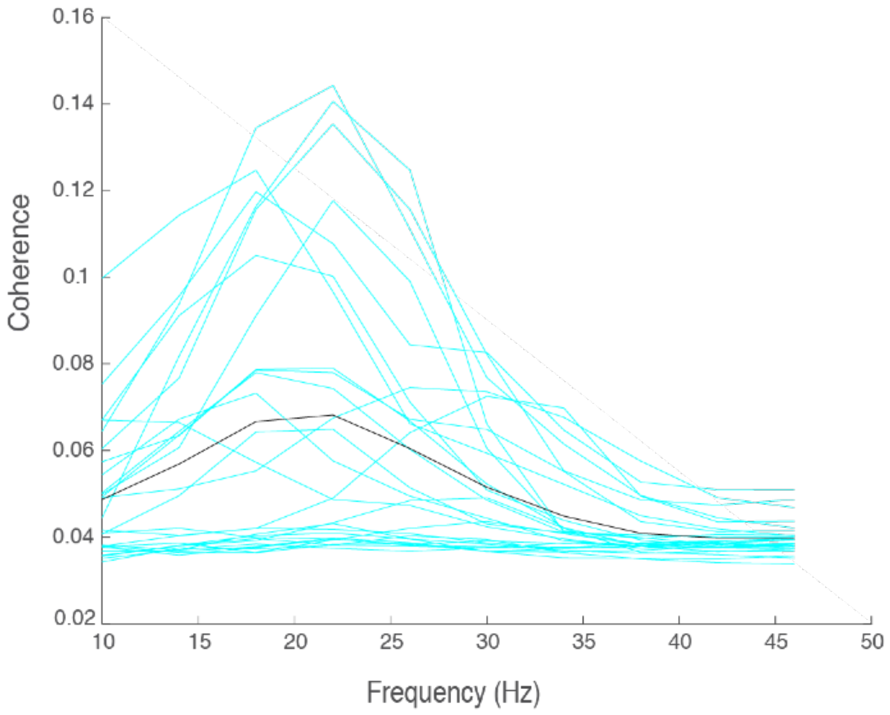

11. Figure C illustrates the grand-averaged topographic maps for EEG power in the beta band (16-36 Hz) before (Pre) and after (Post) training and the contrast between the two conditions (Post-Pre).

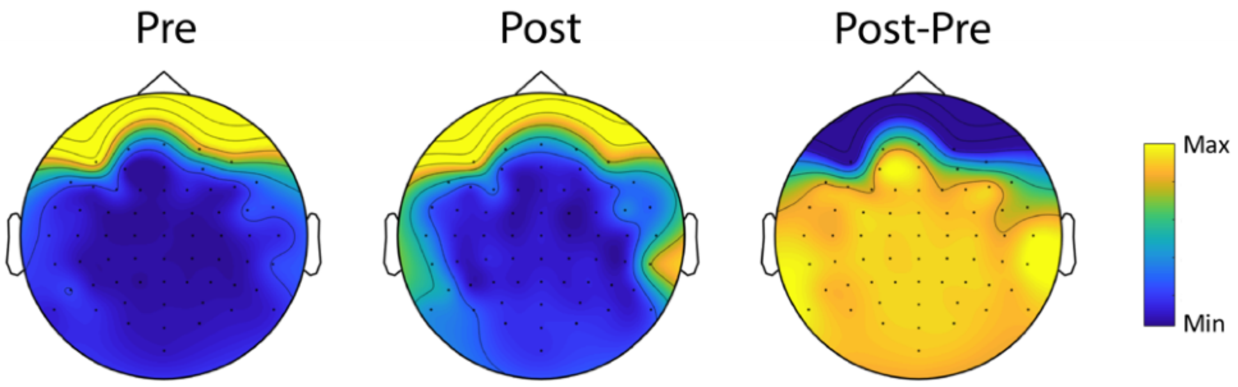

Supplement: Supplementary file 1 — Supplementary Information. [file 41598_2022_10342_MOESM1_ESM.pdf]
